# Origin and Distribution of Daytime Electron Density Irregularities in the Low‐Latitude *F* Region

**DOI:** 10.1029/2020JA028343

**Published:** 2020-09-02

**Authors:** Hyosub Kil, Woo Kyoung Lee, Larry J. Paxton

**Affiliations:** ^1^ The Johns Hopkins University Applied Physics Laboratory Laurel MD USA; ^2^ Korea Astronomy and Space Science Institute Daejeon Korea

**Keywords:** equatorial ionosphere, daytime irregularities, bubbles, fountain effect

## Abstract

Electron density irregularities on the dayside in the low‐latitude *F* region are understood as remnants (or fossils) of nighttime plasma bubbles. We provide observational evidence of the connection of daytime irregularities to nighttime bubbles and the transport of the daytime irregularities by the vertical motion of the background ionosphere. The distributions of irregularities are derived using the measurements of the ion density by the first Republic of China satellite from March 1999 to June 2004. The seasonal and longitudinal distributions of daytime and nighttime irregularities in low latitudes show a close similarity. The high occurrence rate of daytime irregularities at the longitudes where strong irregularities occur frequently at night provides strong evidence of the association of daytime irregularities with nighttime bubbles. Nighttime irregularities are concentrated in the equatorial region, whereas daytime irregularities spread over broader latitudes. The seasonal and longitudinal variation of the latitudinal spread of daytime irregularities is consistent with the morphologies of plasma density and vertical plasma velocity. The zonal wave number 4 pattern, which corresponds to that in plasma density, is identified in the distribution of daytime irregularities. These observations lead to the conclusion that the morphology of daytime irregularities in the low‐latitude *F* region is dominated by the morphology of bubbles at night and the ionospheric fountain process on the dayside.

## Key Points

- The seasonal and longitudinal distributions of daytime and nighttime irregularities in low latitudes are similar
- Zonal wave number 4 patterns in daytime irregularity distribution, plasma density, and vertical velocity show similar behavior
- The morphology of daytime irregularities is determined by the morphology of bubbles at night and fountain process on the dayside

## 1 Introduction

Electron density irregularities in the low‐latitude *F* region are understood in association with ionospheric turbulences accompanied by plasma bubbles. After sunset, the formation of a steep gradient in the vertical electron density and the upward motion of the ionosphere make the bottomside of the *F* region unstable to the generalized Rayleigh‐Taylor instability (Huba et al., 2008; Kelley, 1989; Sultan, 1996). The vertical growth of perturbations on the bottomside results in the transport of low‐density plasma from the bottomside to the topside, which produces plasma depletions (bubbles) on the topside (Kelley et al., 1976; Ossakow & Chaturvedi, 1978; Woodman & La Hoz, 1976). Bubbles decay rapidly after sunrise by photoionization, but some of them survive in the course of a day and become the sources of daytime irregularities. The detection of daytime irregularities at the locations where bubbles had developed the previous night supports the interpretation that daytime irregularities are remnants or fossils of nighttime bubbles (Huang et al., 2013; Kil et al., 2019).

The connection of daytime irregularities to nighttime bubbles is identified by tracing the history of daytime irregularities (Huang et al., 2013; Kil et al., 2019). However, case studies of several events are not yet sufficient to determine the origin of daytime irregularities. Along with the tracing of the history, we can infer the connection of daytime irregularities to nighttime bubbles by examining their occurrence statistics. If daytime irregularities are fossils of nighttime bubbles, the morphology of daytime irregularities would be similar to that of nighttime irregularities. Our study uses this method to determine the origin of daytime irregularities.

One of distinguishing characteristics of daytime irregularities is their latitudinal distribution. Irregularities are concentrated near the magnetic equator just after sunrise, but the concentration shifts to higher latitudes as time progresses (Kil et al., 2019). The morphology of daytime irregularities resembles the formation of the ionization trough at the magnetic equator and the ionization crest at around ±15° magnetic latitudes by the fountain effect (Hanson & Moffett, 1966). Because fossil bubbles would experience the same fountain process, the latitudinal spread of daytime irregularities is assumed to be related to the upward motion of the ionosphere on the dayside (Kil et al., 2019). We validate this hypothesis by comparing the distributions of irregularities, plasma density, and vertical plasma motion.

Two hypotheses that we validate in this study are as follows: (1) Daytime irregularities are fossils of nighttime bubbles, and (2) fossil bubbles are transported to higher latitudes by the fountain effect. For this purpose, we derive the seasonal and longitudinal distributions of irregularities using the measurements of the ion density by the first Republic of China satellite (ROCSAT‐1) from March 1999 to June 2004. The observational data and the detection method of irregularities are described in section 2. Observational results and discussion are presented in section 3. Conclusions are given in section 4.

## 2 Description of Data and Irregularity Detection

ROCSAT‐1 (later renamed as Formosat‐1) was a low‐earth (altitude: 600 km) and low‐inclination (35°) orbit satellite operated by Taiwan from 27 January 1999 to 17 June 2004. This study derives the distribution of irregularities by applying the method used by Kil et al. (2019). This method detects irregularities using the parameter *S* defined as

$$S={\left[\frac{1}{n-1}\sum_{i=0}^{n-1}{\left(\log_{10}N_{i}-L_{i}\right)}^{2}\right]}^{1/2}$$

Here *N*_*i*_ is the electron density, *L*_*i*_ is the linear fit of log_10_*N*_*i*_, and *n* is the number of data points. We have chosen 10 for *n*, but the occurrence statistics of irregularities is not sensitive to *n*. The dependence of the irregularity detection on the density can be minimized by normalizing the density or using the logarithm of the density. Here we use the logarithm of the density provided every 1 s. The spatial resolution of the measurement is ~8 km.

**Figure 1:**
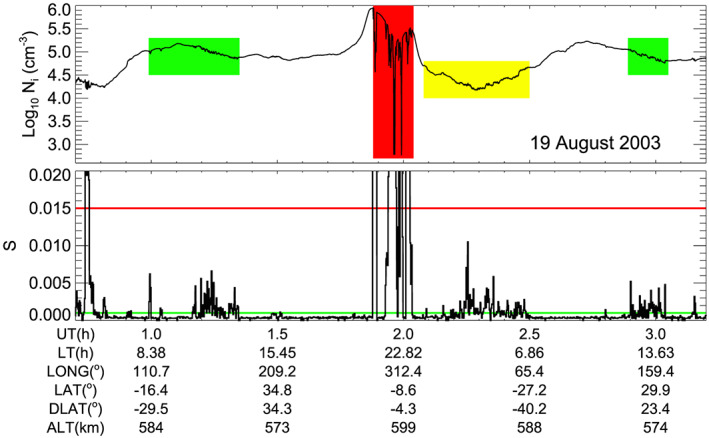
Detection of irregularities from ROCSAT‐1 observations. Typical nighttime bubbles and small amplitude irregularities at night are indicated with red and yellow shadings, respectively. Irregularities detected on the dayside are indicated with green shadings. Nighttime bubbles can be detected using the *S* value of 0.015 (red line), but other irregularities are not detected using this threshold. We use an *S* value of 0.001 (green line) for the detection of small amplitude irregularities.

We illustrate the detection of irregularities with an example shown in Figure 1. Typical nighttime bubbles are indicated with a red shading. They are detected at premidnight in the equatorial region. Small amplitude irregularities, indicated with a yellow shading, are detected at postmidnight. Irregularities detected on the dayside are indicated with green shadings. The base value of *S* at quiet regions is about 0.0005. Both daytime and nighttime irregularities can be detected using a threshold greater than this value. The red line (*S* = 0.015) indicates the threshold used in previous studies for the detection of nighttime bubbles (Kil et al., 2009a; Su et al., 2006). However, the irregularities in yellow and green shadings are invisible when this threshold is used. Our study uses *S* = 0.001 (green line) for the detection of daytime irregularities.

Communication/Navigation Outage Forecast System (C/NOFS) and Swarm satellite data are also used for a case study. The C/NOFS satellite was launched on 16 April 2008 into low inclination (13°) and elliptical (400–700 km) orbit and ended on 28 November 2015. Coupled Ion‐Neutral Dynamics Investigation (CINDI) is part of the payload for the C/NOFS program. We will use the measurements of the ion density by CINDI on 14 December 2013. The data sampling cadence is 1 s.

Swarm is the European Space Agency's mission comprised of three satellites. The satellites were launched on 22 November 2013 into a near circular orbit with an orbital inclination of 87.5° at an altitude of 470 km for two satellites and of 88° at an altitude of 530 km for one satellite. We use the measurements of the electron density by the Langmuir Probe instrument on board the Swarm‐Alpha (Swarm‐A) satellite. The data sampling cadence is 0.5 s. The Swarm data are used only for 14 December 2013 to compare with the CINDI data.

## 3 Observational Results and Discussion

**Figure 2:**
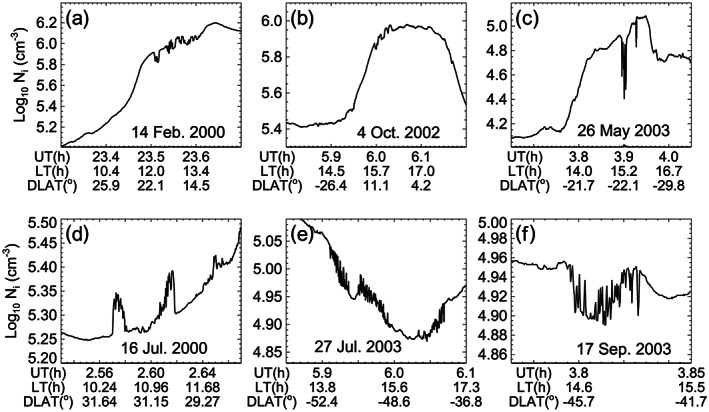
Various structures of daytime irregularities identified from ROCSAT‐1 observations.

Various structures of irregularities occur during daytime. Some examples are presented in Figure 2. Typical forms of daytime irregularities are shown in Figures 2a and 2b. Severe plasma depletions as observed at night do not appear on the dayside because of the rapid refilling of deep plasma depletions by the photoionization. The morphological characteristic of nighttime bubbles (plasma depletion with respect to ambient plasma) remains in the morning, but this characteristic diminishes with time. The examples in Figures 2c–2f are anomalous forms of irregularities that are rarely detected. The irregularities in Figure 2c maintain the morphology of nighttime bubbles, although they are detected several hours after sunrise (15 hr LT). Blob‐like structures appear in Figure 2d, and quasi‐periodic and high‐frequency density fluctuations appear in Figure 2e. The irregularities in Figures 2e and 2f are detected at 40–50°S dip latitudes. We postpone the investigation of conditions for the development of those anomalous irregularities to future work.

**Figure 3:**
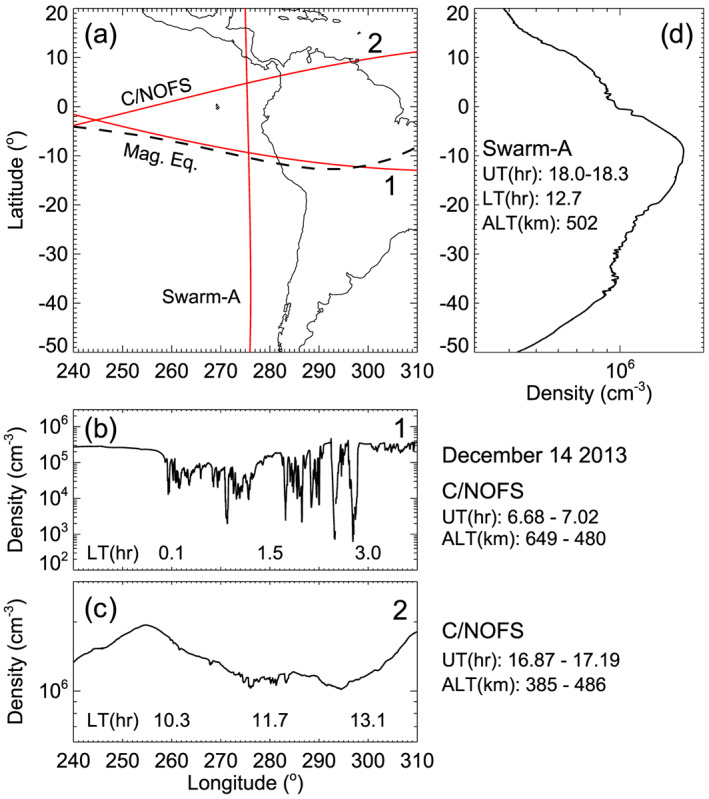
Detection of daytime irregularities at the longitude where bubbles had developed at night. (a) C/NOFS and Swarm‐A orbits. (b and c) Measurements of the plasma density by C/NOFS at night (orbit 1) and on the dayside (orbit 2). (d) Measurements of the plasma density by Swarm‐A on the dayside.

Two distinguishing characteristics of daytime irregularities identified from ROCSAT‐1 observations are their association with nighttime bubbles and the preferential occurrence of daytime irregularities away from the magnetic equator (Kil et al., 2019). Prior to the presentation of the occurrence statistics of daytime irregularities, we demonstrate these characteristics using the observations of the plasma density by Swarm‐A and C/NOFS satellites on 14 December 2013. Satellite orbits on that day are shown on the map in Figure 3a. The measurements of the plasma density along those orbits are presented in Figures 3b–3d. Bubbles have developed at night at 260–300°E longitude, as we can identify from the C/NOFS observation on orbit 1. Irregularities are detected on the consecutive C/NOFS orbits in that longitude region. The observation on orbit 2 is provided to show the occurrence of irregularities on the dayside (~11.7 hr LT). The Swarm‐A observation on the dayside (12.7 hr LT) is made about an hour after the C/NOFS observation on orbit 2. Note the detection of irregularities around ±15° magnetic latitudes, but the absence of irregularities at the magnetic equator in the Swarm‐A observation. Using the occurrence statistics, we assess whether this is a common characteristic of daytime irregularities.

**Figure 4:**
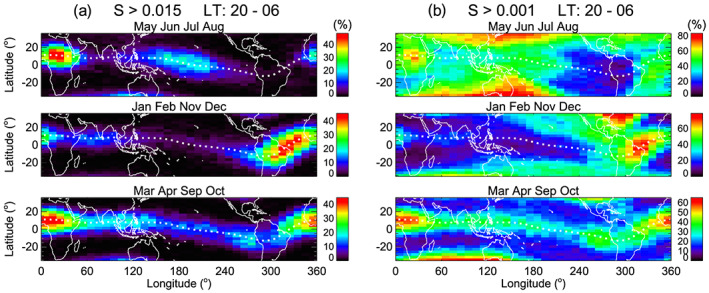
Seasonal and longitudinal distributions of irregularities at night (20–06 hr LT) derived using the thresholds (a) *S* = 0.015 and (b) *S* = 0.001.

To identify the connection of daytime irregularities to nighttime bubbles, we first investigate the morphology of nighttime irregularities. Figure 4 presents the distributions of nighttime (20–06 hr LT) irregularities obtained using the detection thresholds (a) *S* = 0.015 and (b) *S* = 0.001. Months are divided into three seasons: June solstice (May–August), December solstice (January, February, November, and December), and equinox (March, April, September, and October). The maps in Figure 4a represent the typical distributions of nighttime bubbles (or large amplitude irregularities). Because the distribution of nighttime bubbles has already been reported by many studies (Burke et al., 2004; Gentile et al., 2011; Huang et al., 2001; Kil & Heelis, 1998; Kil, Heelis, et al., 2009a; Maruyama & Matuura, 1984; McClure et al., 1998; Stolle et al., 2006; Su et al., 2006; Tsunoda, 1985), we refer the discussion of this topic to those references. The distributions of nighttime irregularities in Figure 4b are somewhat different from those shown in Figure 4a because of the high occurrence rate of small amplitude irregularities in midlatitudes. However, the morphologies in the equatorial region are similar in Figures 4a and 4b.

The seasonal and longitudinal behavior of irregularities in midlatitudes in Figure 4b is similar to that of medium‐scale traveling ionospheric disturbances (MSTIDs). The occurrence rate of MSTIDs is higher during solstices than during equinoxes (Ding et al., 2011; Kil & Paxton, 2017; Martinis et al., 2010; Shiokawa et al., 2003). The highest occurrence rate of MSTIDs in the Asian sector during June solstices is also consistent with the distribution of irregularities in Figure 4b. In midlatitude during December solstices, the occurrence of irregularities is pronounced over North America around 300°E longitude. Because this region coincides with the region where conjugate photoelectrons produce perturbations in the ionosphere and thermosphere (Kil et al., 2020), some of the irregularities in that region may be attributed to conjugate photoelectrons. We have examined the distributions of irregularities by varying the detection threshold and the LT range. With the increase of the value of the detection threshold, the occurrence rate of irregularities decreases rapidly poleward of ±15° magnetic latitudes, and the distributions of irregularities become closer to those in Figure 4a. The distributions are not sensitive to the selection of the LT range. The comparison of Figures 4a and 4b indicates that the irregularities associated with bubbles are much more intense than those associated with MSTIDs or conjugate photoelectrons.

**Figure 5:**
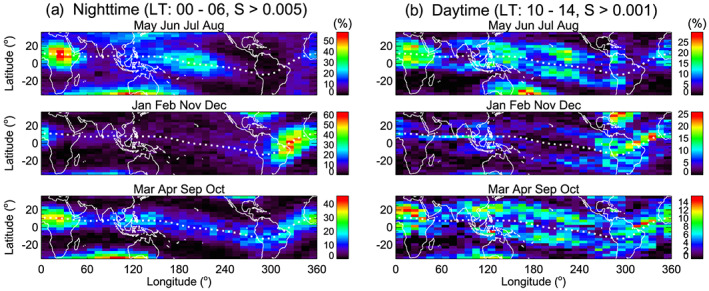
Comparison of the distributions of (a) postmidnight and (b) daytime irregularities. Nighttime and daytime irregularities are detected using the *S* values of 0.005 and 0.001, respectively.

The distributions of irregularities at postmidnight (00–06 hr LT) derived using the threshold *S* = 0.005 are shown in Figure 5a. We present the distributions of nighttime irregularities derived using different detection thresholds and LT ranges in Figures 4a, 4b, and 5a to provide an opportunity to compare them with daytime irregularities. Large amplitude irregularities detected at late night are assumed to have a higher chance of surviving on the dayside, but we cannot specify which irregularities persist on the dayside. Using Figures 4a, 4b, and 5a, we emphasize that the morphology of nighttime irregularities in low latitudes does not vary much depending on the selection of LT and detection threshold. The morphology of irregularities established at premidnight dominates the morphology at postmidnight and even on the dayside.

The distributions of daytime irregularities are shown in Figure 5b. They are derived using the detection threshold *S* = 0.001 for the data at 10–14 hr LT. Daytime irregularities are mostly confined to low latitudes by the disappearance of nighttime irregularities in midlatitudes. This observation provides a clue for the source of daytime irregularities in low latitudes. The confinement of daytime irregularities to low latitudes indicates that large amplitude irregularities at night survive on the dayside. In other words, bubbles are the primary sources of daytime irregularities. Some of the daytime irregularities in low latitudes may be produced by MSTIDs. However, the fraction of daytime irregularities associated with MSTIDs would be small considering the confinement of daytime irregularities to low latitudes.

Comparing Figures 5a and 5b, we can identify similarities in the seasonal and longitudinal distributions of daytime and nighttime irregularities. The peaks of their occurrence rates occur in the African sector during June solstices and equinoxes and in the Atlantic sector during December solstices. Here we note two differences. First, nighttime irregularities are concentrated in the equatorial region with the peak occurrence rate at the magnetic equator, whereas daytime irregularities spread over broader latitudes with the peak occurrence rate often outside the magnetic equator. Second, the longitudinal patterns of daytime and nighttime irregularities are somewhat different during June solstices. During June solstices, the occurrence rate of daytime irregularities is pronounced around 120°E longitude, but its matching feature is not obvious in the distribution of nighttime irregularities. In Figure 4b, small amplitude irregularities occur in broad longitude regions at night during June solstices. Some of them may contribute to the daytime irregularities around 120°E longitude.

The longitudinal distribution of daytime irregularities during June solstices is similar to the wave number 4 pattern in plasma density driven by nonmigrating zonal wave number 3 tide (DE3) (Forbes et al., 2006, 2008; Hagan & Forbes, 2002; Oberheide et al., 2006). The *E* region dynamo is modulated by DE3 and by which the zonal wave number 4 pattern in plasma density and vertical plasma motion develop (England et al., 2006; Fejer et al., 2008; Immel et al., 2006; Kil et al., 2008; Lin et al., 2007; Liu & Watanabe, 2008; Lühr et al., 2008; Pedatella et al., 2008; Ren et al., 2008; Scherliess et al., 2008; Wan et al., 2008). Because this phenomenon is the manifestation of the control of the latitudinal and longitudinal distribution of plasma by the fountain effect, the formation of the zonal wave number 4 pattern in the distribution of daytime irregularities provides strong evidence of the significant role of the fountain effect in the formation of the morphology of daytime irregularities.

**Figure 6:**
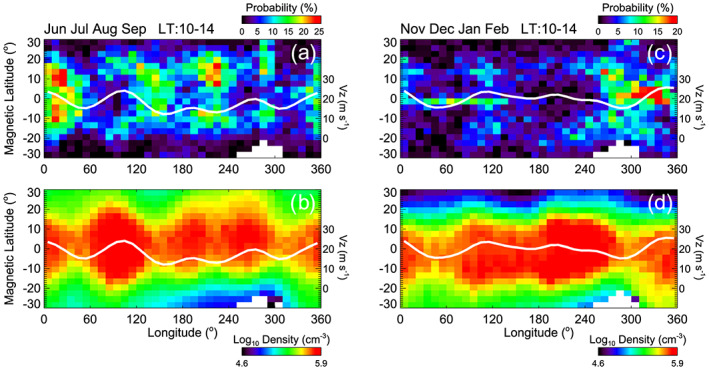
Comparison of the distributions of daytime irregularities and plasma density during the periods of (a, b) June–September and (c, d) November–February. Irregularities are detected using the *S* value of 0.001. The vertical ion velocity is shown with white lines.

In Figure 6, the distributions of daytime irregularities, plasma density, and vertical plasma velocity are compared for two periods of the year: (a, b) June–September and (c, d) November–February. For this comparison, we use the data at 10–14 hr LT. The vertical plasma velocity shown by a white line is the empirical model (Kil et al., 2009b) driven under the condition of the *F*10.7 index between 130 and 200 solar flux units (sfu, 1sfu = 10^−22^ W/m^2^/Hz). June–September is the period during which both DE3 and the ionospheric wave number 4 pattern are pronounced, and November–February is the period during which the bulges of plasma density occur at three longitude regions (Kil & Paxton, 2011). The comparison of Figures 6a and 6b reveals the similarity in the morphologies of daytime irregularities and plasma density. The troughs of the occurrence rate at the magnetic equator are formed at the longitudes where the bulges of plasma density are formed. Both phenomena are explained by the transport of equatorial irregularities and plasma to higher latitudes by the fountain effect. The vertical ion velocity is consistent with the morphologies of irregularities and plasma density.

The correlated behavior of irregularities, plasma density, and ion velocity is also observed during the period of November–February (Figures 6c and 6d). During this period, daytime irregularities are pronounced in the American‐Atlantic sector (270–340°E), consistent with the distribution of nighttime irregularities. The concentration of daytime irregularities and plasma near the magnetic equator around 300°E longitude can be explained by the small upward plasma velocity in that region. However, daytime irregularities are seen to be concentrated to the equatorial region in the whole American‐Atlantic sector, although the vertical plasma velocity varies with longitude. This longitude region is characterized by the negative magnetic declination. In this region, eastward winds compete with summer‐to‐winter (south‐to‐north) winds for control of the plasma motion along magnetic field lines during December solstices (Lee et al., 2018). We interpret the concentration of daytime irregularities to the equatorial region as related to the suppression of the fountain process by equatorward wind components.

## 4 Conclusions

Two hypotheses raised by Kil et al. (2019) regarding the origin and distribution of daytime irregularities in low latitudes are validated using the ROCSAT‐1 data taken from March 1999 to June 2004. The hypothesis that daytime irregularities are fossils of nighttime bubbles is supported by the observation of the high occurrence rate of daytime irregularities at the longitudes where strong irregularities develop at night. The notable difference between the morphologies of daytime and nighttime irregularities is in the latitudinal distribution. Nighttime irregularities are concentrated near the magnetic equator, whereas daytime irregularities are spread over broader latitudes. The latitudinal distribution of daytime irregularities shows a good agreement with the distribution of plasma driven by the vertical plasma motion. The observation of the zonal wave number 4 pattern in the distribution of daytime irregularities, which is consistent with the zonal wave number 4 patterns in plasma density and vertical plasma motion, provides strong evidence of the latitudinal redistribution of fossil bubbles by the fountain effect. Thus, the morphology of nighttime irregularities and vertical plasma motion are two dominant factors that determine the morphology of daytime irregularities in low latitudes.

## Data Availability

The ROCSAT‐1 and CINDI data are available from NASA heliophysics Data Portal (https://heliophysicsdata.sci.gsfc.nasa.gov/websearch/dispatcher). Swarm data are available at the website (https://earth.esa.int/web/guest/missions/esa-operational-eo-missions/swarm).
